# The Usefulness of Evaluating Performance of Activities in Daily Living in the Diagnosis of Mild Cognitive Disorders

**DOI:** 10.3390/ijerph182111623

**Published:** 2021-11-05

**Authors:** Patricia De Vriendt, Elise Cornelis, Wilfried Cools, Ellen Gorus

**Affiliations:** 1 Frailty in Ageing (FRIA) Research Group, Department Gerontology, Vrije Universiteit Brussel, 1090 Brussel, Belgium; ellen.gorus@vub.be; 2 Department Occupational Therapy, Artevelde University of Applied Sciences, 9000 Ghent, Belgium; elise.cornelis@arteveldehs.be; 3 Department Occupational Therapy, University Ghent, 9000 Ghent, Belgium; 4 Interfaculty Center Data Processing and Statistics, Vrije Universiteit Brussel, 1090 Brussel, Belgium; wilfried.cools@vub.be; 5 Department Gerontology and Geriatrics, Universitair Ziekenhuis Brussel, 1090 Brussel, Belgium

**Keywords:** everyday functioning, activities of daily living, dementia, mild cognitive impairment, assessment

## Abstract

The Assessment of Activities of Daily Living (ADL) is paramount to ensure the accurate early diagnosis of neurocognitive disorders. Unfortunately, the most common ADL tools are limited in their use in a diagnostic process. Hence, we set out to validate a tool to evaluate basic (b-), instrumental (i-), and advanced (a-) ADL called the Brussels Integrated Activities of Daily Living Inventory (BIA). At the geriatric day hospital of the University Hospital Brussels (Belgium) older persons (65+) labelled as Cognitively Healthy Persons (CHP) (*n* = 47), having a Mild Cognitive Impairment (MCI) (*n* = 39), and having Alzheimer’s disease (AD) (*n* = 44) underwent a diagnostic procedure for neurocognitive disorders. Additionally, the BIA was carried out. An exploration using both (cumulative) logistic regressions and conditional inference trees aimed to select the most informative scales to discriminate between the HCP, persons with MCI and AD. The distinction between CHP and MCI and between MCI and AD was moderately successful with the i-ADLs, in addition to age. Therefore, it is advisable to conduct a multidomain assessment in which the i-ADL could serve as non-invasive and non-time-consuming screening, while the BIA might be useful for diagnostics and disease management.

## 1. Introduction

Dementia is a progressive clinical syndrome that affects cognitive abilities and behavior, significantly interfering with older persons’ autonomy and ability in everyday functioning [1,2,3,4]. Today, it is estimated that 47 million people live with dementia worldwide. Due to the ageing of the population, this number is expected to increase dramatically, since advancing age is the most important risk factor [5]. Despite many efforts, dementia cannot be resolved or cured. Nevertheless, a diagnosis of dementia offers opportunities for psychosocial interventions, which focus on improving and optimizing functioning and quality of life, preferably already at an early stage [6]. Therefore, an early diagnosis is of utmost importance. The concept of Mild Cognitive Impairment (MCI) has been developed to capture the early phase of cognitive deterioration and is considered as the transitional stage between normal cognitive functioning and early dementia [7]. The distinction between MCI and dementia can be made based on the extent to which cognitive decline interferes with everyday functioning in activities of daily living (ADLs). In dementia, cognitive impairment decreases independence in everyday functioning, while in MCI, individuals remain autonomous; although subtle problems may already occur in complex ADLs, they may take more time or may be performed less efficiently [8,9,10]. The level of the loss of autonomy in performing ADLs is a significant predictor of future decline and dementia onset in both cognitively healthy older persons and persons with MCI, even after controlling for a variety of relevant variables such as age, sex, education, mood, global cognition and physical burden [11]. Therefore, the assessment of ADLs is paramount to determine the degree of impairment in everyday functioning and to underpin the accurate diagnostic classification in cognitive disorders, taking into account the ability to perform ADLs increases the specificity and sensitivity of the diagnosis [12].

Several typologies and, accordingly, assessment tools are available to describe and evaluate ADLs, but the concept of Reuben et al. [13] seems the most appropriate to use for cognitive problems in a geriatric population [14]. According to Reuben et al. [13], three levels of ADLs exist, organized according to the difficulty, complexity, and vulnerability of cognitive decline. Firstly, basic (b-) ADLs, traditionally described in the Katz-Index [15], are activities that meet basic physiological and self-maintenance needs such as personal hygiene, dressing, and eating. Secondly, instrumental (i-) ADLs include, as described in the Lawton-Scale [16], activities which are essential to preserve autonomous living such as cooking, managing medication and grocery shopping. In this sense, these two levels of ADLs are generally used in practice and research. However, the third level is often lacking in the literature and measurement tools, i.e., the advanced (a-) ADLs, also known as complex or extended ADLs. The a-ADLs cover activities that are volitional and influenced by cultural and motivational factors, expressing a personal engagement in satisfying activities. These a-ADLs go beyond what is needed to be independent. Examples of a-ADLs are leisure, self-development activities, or (semi-) professional work. Since a-ADLs are the most complex activities and require excessive neuropsychological organization, these a-ADLs have proven to be useful in diagnosing cognitive disorders. In fact, the first observable limitations in ADLs due to cognitive decline are seen in a-ADLs [13,14].

When these three levels of ADLs are related to cognitive decline, a functional continuum emerges; in contrast to cognitively healthy older persons, persons with MCI gradually develop subtle but disturbing functional problems (firstly in a- and later in i-ADLs). In persons with dementia, functional problems on all levels of ADLs occur, including b-ADLs in severe dementia. Therefore, a stepwise hierarchical decline of functional abilities can be observed with b-ADLs affected after i- and a-ADLs. Functional decline occurs over the course of MCI and cumulative changes accompany the conversion to dementia [17,18,19].

Many attempts were made to find sensitive, easy-to-administer and inexpensive assessments to evaluate ADLs. Despite the multitude of available report-based evaluations, most evaluations use an unclear and variable understanding of the concept of everyday activities, show several methodological problems such as poor psychometric properties, dichotomous scoring (pass or fail), not addressing underlying causes of impairment (for an overview see De Vriendt et al. [20]).

Therefore, based on all the available evidence, and to overcome existing shortcomings in assessment, our research group set out to develop and validate a comprehensive ADL tool, called the Brussels Integrated Activities of Daily Living Inventory (BIA). It is a compilation of the a-ADL tool [21,22] and the b- and i-ADL tool [23], which are developed and validated separately and in different clinical samples. The a-ADL tool was developed since a tool that measured complex activities was missing. The b- and i-tool comprises an adaptation of the existing ADL evaluations, the Katz [15] and the Lawton Scale [16] but uses the same architecture as the a-ADL tool.

So, the BIA 64 ADLs (6 b-, 9 i-, and 49 a-ADLs) is innovative since it only takes those activities into account that are relevant for the person, in other words the BIA evaluates what somebody does in his or her current environment. It is designed as a self- or informant-reported questionnaire for a patient and/or proxy and is performance based, since it focuses on what a person actually does in his or her current environment. By asking the persons to tell about his or her daily activities and to describe the current performance (and eventually the experienced or observed problems) it is possible to score the quality of performance by using the rating system adopted from the performance qualifiers of the International Classification of Functioning, Disability and Health (ICF) of the WHO in 2001, which is considered as the standard for assessment. Additionally, the underlying cause of impairment can be rated. Based on the previous information, a ‘global disability index’ (DI) is calculated, taking into account the total number of activities found relevant, the number of activities that are limited, and the severity of the limitations according to the ICF scores. A ‘cognitive disability index’ (CDI), ‘physical disability index’ (PDI), an ‘intrapersonal disability index’ (IDI), a ‘social disability index’ (SDI) or a ‘physical environmental disability index’ (EDI) can be computed, considering exclusively the activities that are limited because of cognitive, physical, intrapersonal, social or physical environmental problems, respectively. The activities in which the limitation is, for instance, partly due to both physical and cognitive reasons are included in both indices. As an example, the cognitive index reflects the proportion of limited activities due to cognitive reasons, multiplied by the severity of the limitations, relative to the total number of activities. The indices are expressed as percentages, with lower scores indicating less disability. The a-ADL and the b- and i-ADL are already well investigated for reliability and discriminative validity [14,21,22,23,24].

The objective of this study was to demonstrate the functional decline on the three levels of ADL, to test the hypothesis that this functional decline in ADLs can discriminate between cognitively healthy persons and persons with MCI and dementia, and, finally, to evaluate how the BIA, as a comprehensive tool encompassing the three levels of ADL, can efficiently be used to discriminate between patients with different levels of cognitive impairment in the same clinical sample.

## 2. Materials and Methods

### 2.1. Participants and Procedure

Three groups of community-dwelling, older persons (65 years or older) were consecutively recruited at the geriatric day hospital of an academic teaching hospital (UZ Brussel, Belgium): cognitively healthy persons (CHP), patients with MCI and patients with mild Alzheimer disease (AD). All patients underwent a standardized, multidisciplinary diagnostic procedure consisting of a physical and neurological examination, clinical history taking, functional evaluation, neuropsychological assessment, an extensive laboratory blood testing, and imaging of the brain by CT or MRI scan. Diagnosis was based on a clinical judgment by consensus of the multidisciplinary team. Patients were referred for the first time for a cognitive diagnosis. They were labelled with MCI in accordance with the criteria of the International Working Group on diagnostic criteria for amnestic MCI [7], or with AD in accordance with the criteria of the National Institute of Neurological and Communicative Disorders and Stroke and the Alzheimer’s Disease and Related Disorders Association (NINCDS-ADRDA) [4]. CHP represented geriatric patients who visited the geriatric day hospital for diagnosis or treatment of conditions other than cognitive disorders (e.g., osteoporosis), and thus were recruited separately from the patients with a cognitive referral. They were considered cognitively healthy if a significant impairment in cognition was absent, operationalized by a score >25/30 on the Mini-Mental State Examination (MMSE) [25], a score >79/105 on the Cambridge Cognitive Test (CAMCOG-R), a score >17/27 on the CAMCOG memory section [26], and the absence of a self- or informant-based complaint or history of functional or cognitive deficits suggestive of MCI or AD. For all participants, exclusion criteria were acute pathology, taking antidementia drugs, having sensory or communicative impairments that prohibited them from performing the assessments, and a history of major psychiatric illness or any other pathology of the central nervous system other than MCI or AD (e.g., stroke, epilepsy). An additional exclusion criterion for patients with MCI and AD was the absence of a reliable informant, in order to control over- or underestimation of functional abilities in the assessment.

### 2.2. Measurements

During the consultation at the day hospital, the assessments of cognitive functions were completed. Global cognitive functioning was assessed by the Mini Mental State Examination (MMSE) [25]. The MMSE is a brief cognitive screening covering six domains: language, orientation, attention and calculation, registration, praxis, and recall. It provides a total score of 30 with lower scores indicating greater impairment. A score below 24 refers to major cognitive problems. Additionally, the CAMCOG-R [26] was administered. The CAMCOG consists of 59 items and several subscales: orientation, expressive and comprehensive language, memory, attention, praxis, calculation, abstraction, and perception. This test gives a total score of 105 with lower scores indicating greater impairment. A score below 80 is seen as indicative of major cognitive problems. The original cut-off score of 79/80 showed a sensitivity of 92% and a specificity of 96% for the diagnosis of dementia in the discrimination from normal controls. The memory subscale score of 18/27 achieved a sensitivity of 78% and a specificity of 74% for predicting the conversion to AD from MCI, with an accuracy of 0.83.

In addition, the BIA was administered, including the b-ADL part evaluating 6 b-ADLs (e.g., washing and dressing oneself), the i-ADL part encompassing 9 i-ADLs (e.g., shopping, preparing meals), and the a-ADL part evaluating 49 a-ADLs (e.g., crafts, using complex technologies, self-educational activities). A list of all ADLs is available as Supplementary Material Table S1: the items of basic, instrumental, and advanced Activities of Daily Living of the Brussels Integrated Activities of Daily Living Inventory. The assessment starts by asking persons to share their daily activities and to describe the current performance, and thus the experienced or observed (in case of proxies) problems. The tool suggests thinking back to the years since retirement. If an activity is performed by the patient it is included in the scoring. By asking the persons to describe the current performance (and eventually the experienced or observed problems) it is possible to score the quality of performance. To do this, we adopted the ICF scoring system. The ICF uses a five-point scale, ranging from 0 (no difficulty to perform) to 4 (complete difficulty). If performance scores are >0, the underlying cause (cognitive or physical) for the limitation is rated and all different influencing components of daily functioning are disentangled. The assignment of a cause is dichotomous: ‘yes’ when a cause is present and ‘no’ when a cause is absent. Based on the gathered information, specifically for this population, a ‘global disability index’ (DI) is calculated, taking into account the total number of activities found relevant, the number of activities that are limited, and the severity of the limitation according to the ICF scores. Next to the DI a ‘cognitive disability index’ (CDI) and a ‘physical disability index’ (PDI) is calculated, considering exclusively those activities that are limited because of cognitive (e.g., memory, attention) or physical (e.g., loss of strength, mobility) problems, respectively. Activities in which the limitation is partly due to physical and partly due to cognitive reasons are included in both indices. The indices are expressed as percentages, with lower scores indicating less disability.

In this study the DIs, CDIs, and PDIs are calculated for b-, i-, and a-ADLs. For all assessments of the BIA, self-reporting was used for CHP and informant-reporting was used for MCI and AD. The a-ADLs and the b- and i-ADLs showed a satisfactory reliability and discriminative validity [14,21,22,23,24].

Additionally, demographic data were collected: age, gender, and years of education.

### 2.3. Statistical Analysis

Firstly, demographic characteristics, cognitive data, and BIA data were analyzed. The differences between CHP, MCI, and AD were evaluated by one-way ANOVA for continuous variables or chi-square analysis for categorical variables. To localize the differences between CHP versus MCI, MCI versus AD, and CHP versus AD, Bonferonni post hoc group-by-group testing was performed. These statistical analyses were performed with IBM SPSS (IBM Corp. Released 2020. IBM SPSS Statistics for Windows, Version 27.0. Armonk, NY, USA) with an α-level set two-sided at *p* < 0.05 for all analysis.

Secondly, the severity of impairment was analyzed in relation to the BIA data, controlling for the demographic characteristics where relevant. The correlations among the indices were considered first, with a follow-up using a partial least squares attempt to combine the indices and conditional inference trees, which were then used to determine which of the indices would dominate the prediction of the different groups. The individual indices were finally evaluated as predictors combined with demographic information, using logistic regression to distinguish between the CHP and MCI, and between the MCI and AD. Both were also combined with a cumulative logistic regression. Note that model selection was performed based on the Akaike Information Criterion (AIC), with great care to avoid multicolinearity that was expected because of the strong correlations among indices.

### 2.4. Ethical Considerations

The Ethical Committee of the UZ Brussel approved this study (B.U.N. 143201523678). All data were collected in accordance with the ICH-GCP guidance and the Helsinki Declaration. Participants and informants gave written informed consent.

## 3. Results

One hundred and thirty participants (86 female, 44 male; 47 CHP, 39 MCI, 44 AD) were included, with a mean age of 80.69 years (5.48; 68–96). Although the demographics for the different groups, CHP, MCI, and AD, were largely overlapping, significant differences were noted for the age and educational level of the participants (Table 1). Table 1 also shows the results of the MMSE, CAMCOG total score, CAMCOG memory score and BIA indices. As expected, significant differences were observed for almost all variables except for some b-ADL indices and the PDI indices. The DI and CDI of b-, i- and a-ADL showed significantly less disability in CHP than in MCI and the latter less than in AD. This is clearly less apparent for the PDI, which gives a more diffuse picture.

After analyzing the data for men and women separately, the results are as follows: for the whole group, only significant differences could be observed for education and the MMSE and CAMCOG total scores (all *p* < 0.05), with men performing better than women. In the diagnostic groups: in the CHP no significant differences were observed; in the MCI only significant differences for education were observed (*p* < 0.05); in the AD group: significant differences for the MMSE and CAMCOG scores were observed (all *p* < 0.05), and again with men performing better than women.

**Figure 1:**
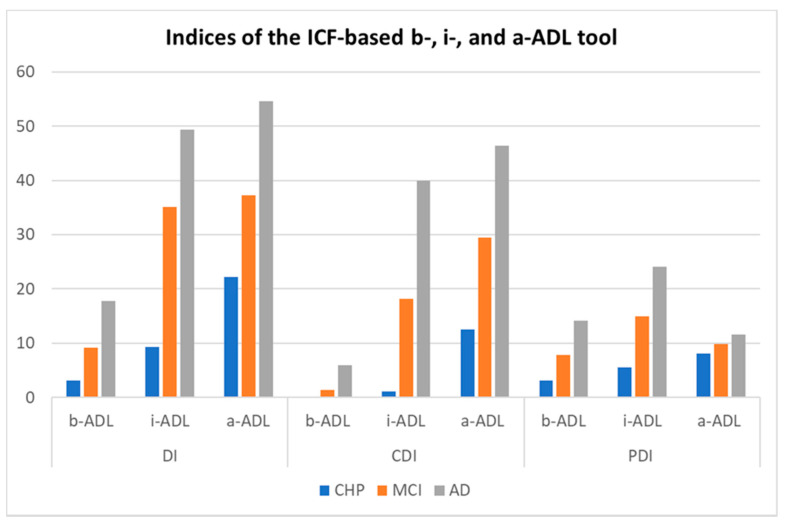
Functioning of cognitively healthy persons (*n* = 47), patients with MCI (*n* = 39) and patients with mild AD (*n* = 44) measured with the BIA indices expressed as percentages. Legend—ICF: International Classification of Functioning, Disability and Health; CHP: Cognitively Healthy Persons; MCI: Mild Cognitive Impairment; AD: Alzheimer’s Dementia; ADL: Activities of Daily Living; b-: basic; i-: instrumental; a-: advanced; DI: Disability Index; CDI: Cognitive Disability Index; PDI; Physical Disability Index. Indices expressed as percentages.

Figure 1 illustrates the ‘functional continuum’ from age-related functional decline, over subtle problems in a-ADL in MCI patients and more pronounced functional problems in dementia. This continuum is clearly visible in the global indices (DI’s) and the cognitive indices (CDI’s) and, as expected, less in the physical indices (PDI’s).

To evaluate how the BIA tool could be used to discriminate between CHP persons with MCI and AD, a cumulative logistic regression was used, preceded by various exploratory steps. While the aim was to combine the various indices as predictors, in combination with gender, education and age, the correlations among these indices were so high (almost a third of the pairwise correlations exceeded 0.68 and some even 0.90) that most available information would be captured by only a few of them. The partial least squares analysis (not included in more detail) suggested that all indices could be combined into just one, potentially two, indices, but at the expense of interpretability. Additionally, the conditional inference trees (not included in more detail) clearly highlighted that after including two indices, most often i-ADL-DI and i-ADL-CDI, the inclusion of other indices did not improve the prediction.

The cumulative logistic regression that focused on the prediction of CHP, MCI, and AD, based on the ADL-ratings, was built as dependent on age. The resulting model is relatively small and, without too much loss, only considers one of the scales. Because of the strong inter-correlations, it is possible, without much loss, to replace one index with another.

The model we propose is selected using the AIC, and, in addition to age, it includes i-ADL-DI and i-ADL-CDI and their interactions. The model first estimates one intercept, distinguishing between CHP and persons with MCI at 8.395 (se = 3.270), and a second intercept, distinguishing between persons with MCI and persons with AD at 11.151 (se = 3.349). The actual probabilities for observing these categories depend on both the i-ADL-DI and i-ADL-CDI, which have strong negative interactions, reflecting that, for a higher probability to score poorly, either probability should be high. The negative estimate for the interaction is −19.955 (5.145) which compensates for the two positive contributions of the main effects of i-ADL-DI with 5.414 (se = 1.517) and i-ADL-CDI with 16.302 (se = 3.487). Age also shows a significant effect, albeit small, with an estimate of 0.087 (se = 0.041), implying a much higher probability of ending up in the AD group as age progresses.

Unfortunately, the predictions remained rather poor. The actual labels (rows) were often predicted as incorrect (off diagonal frequencies) (see Table 2). The overall correctly predicted diagnosis was 63% and ranged from 23.1% for the persons with MCI to 85.1% for the CHP.

**Figure 2:**
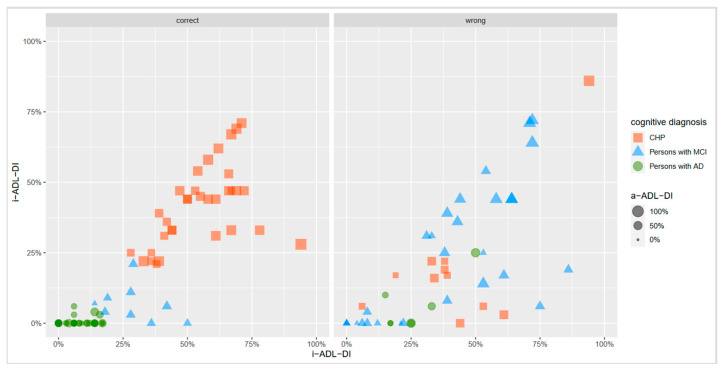
Visualization of the actual BIA data by diagnostic groups. Legend—CHP: Cognitively Healthy Persons; MCI: Mild Cognitive Impairment; AD: Alzheimer’s Dementia; i-ADL-DI: instrumental Activities of Daily Living Disability Index; a-ADL-DI: advanced Activities of Daily Living Disability Index; i-ADL-CDI: instrumental Activities of Daily Living Cognitive Disability Index.

This problem is made clear simply by visualizing the actual data for a few of the scales, as shown in Figure 2. While distinguishing between CHP and persons with AD appeared to be relatively straightforward, for example by using a cut-off for an a-ADL-DI slightly higher than 0.25, the middle category did not separate significantly. Separation was also possible for an a-ADL-CI of around 0.25, or for an i-ADL-CL based on the size. Figure 2 captures three scales: i-ADL-DI on the X-axis, a-ADL-CDI on the Y-axis, and a-ADL-CDI as the third dimension represented by the size. Note that the a-ADL-DI cannot be included jointly with the other two, but can stand by itself, as it largely offers the same information.

## 4. Discussion

In this study, it was the objective to demonstrate the continuum of functional decline and to evaluate how the BIA could discriminate between older persons with different levels of cognitive impairment, CHP, and patients with MCI and AD.

As expected the global and cognitive indices of b-, i- and a-ADLs showed significantly less disabilities in CHP than in MCI, and the latter less than in AD, clearly demonstrating the hierarchical continuum of functional decline. This is clearly less apparent for the PDI, which gives a more diffuse picture. These findings indicate that one should focus on the functional impairment caused by cognitive problems and, moreover, illustrate the importance of making a distinction in the causes of limitations, especially in older patients, in whom physical limitations, which also affect everyday functioning, are commonly observed [23].

ADLs commonly refer to the ability to perform everyday activities and are seen as a measurement of disability and independence in daily life [27]. Daily functioning is complex, resulting in an interplay of different factors requiring an optimal combination of cognitive, motor, and psychological skills, as well as the appropriate environmental conditions. Therefore, a good ADL evaluation should rely on an appropriate differentiation between the causes of underlying limitations in performing ADLs. It is a major challenge and of utmost importance, particularly in older persons, to clarify what causes limited functioning and to determine to what extent functional limitation is due to cognitive limitations and not due to other causes [28]. The BIA is, to our knowledge, the only report-based evaluation that differentiates between the causes of limitations in performing ADLs. The causes of limitations are identified by listening to the stories of the patient or his/her proxy. Based on these stories, the clinician assigns one or more cause(s) of the limitations. It has to be acknowledged that this judgement remains dependent on the quality of the information provided by the patient or his/her proxy. However, it is demonstrated that trained clinicians can accurately judge self and/or informant reports on ADLs [29]. Nonetheless, since the most reported cause of limitations in ADLs is ‘old age’ [30,31], it is important that clinicians carefully explore the reasons of limitations by using probes such as ‘How do you/does (s)he performs this activity?’ or ‘What causes the need for help to perform this activity?’.

The nine indices appeared to be strongly correlated, implying that they shared the most information and, therefore, were largely redundant when combined. When asking which index was most interesting, it appeared that the distinction between CHP and MCI, and the distinction between MCI and AD, was moderately successful when combining the i-ADL-DI and the i-ADL-CDI, in addition to age. It was possible to label most of the CHP correctly. Distinguishing between MCI and AD remained more error-prone. In other words, this study showed that the i-ADLs necessary to live independently, partly in combination with the so-called a-ADLs such as hobbies or sports, are able to differentiate between CHP and those who most likely have a cognitive disorder. The i-ADL seems to be a better predictor, most likely because the ADLs are more consistent, since most -i-ADLs were relevant for each person, aside from some gender issues. This is not the case for the a-ADLs which are more diverse and personal. The a-ADL tool is an amalgam of different activities with different levels of cognitive (and physical) loads. Moreover, it is also possible that persons with cognitive problems already choose activities which are less difficult, meaning that the cognitive decline is less apparent.

It was more difficult to differentiate between mild and moderate-to-severe cognitive problems. It was consistently found that a greater cognitive impairment was associated with increased levels of functional dependency. Several studies showed a significant relationship between everyday functioning and memory [32], executive functioning [33,34], complex reasoning [35], and global cognition [36]. An explanation of the cognitive correlates of functional status found that the variance in daily functioning that could be specifically attributed to cognition was modest. However, in our previous studies, it was shown that executive functioning contributed to the same amount of variance in the limitations in both i- and a-ADLs; this might also explain the fact that i-ADL was a better/stronger predictor than a-ADL. Although it seems disappointing that we could not demonstrate a higher accuracy, the fact that we could isolate the CHP from those with a cognitive problem is clinically interesting, since the patient groups required the same follow-up and disease management.

Other studies also demonstrated that the differentiation between cognitive disorders is challenging [37]. As we know that functional deviations in MCI are situated between those seen in healthy aging and those seen in dementia, an overlap is clear. This implicates that the difference between normal ageing and MCI on the one hand, and MCI and dementia on the other hand, is difficult to distinguish. This is why the debate about the MCI criteria and their operationalization is ongoing. The new version of the Diagnostic Statistical Manual of Mental Disorders makes a differentiation between mild and major neurocognitive disorders (NCD) and demands a stronger weight of independence in ADL. The challenging part is the fact that the functional status varies between persons and that, to date, no normative data exist. From an individual’s point of view, it is the individual slope of decline that is the most important, rather than age-specific norms [38]; ADL tools should allow such a personalized evaluation. However, existing assessment tools do not allow for a ‘personalized’ measurement because it is difficult to reach psychometrical quality since no solution has been found yet to take into account the variability in scoring. Therefore, more high-level statistical strategies should be applied. When taking the BIA as an example, the indices provide a quick and overall representation of a person’s (dis)ability to perform b-, i-, and a-ADLs. The single tools showed good to excellent content, face, construct, and convergent and discriminative validity. Furthermore, the BIA as comprehensive tool demonstrated screening potential. Thus, even within personalized ADL-evaluations, both standardization and personalization are possible and excellent validity should be the main objective.

Although the results of this study show that, for now, the BIA cannot be used as a standalone diagnostic tool for cognitive disorders, it still has an important place within an all-embracing, multidisciplinary, diagnostic research area including at least medical, cognitive, psychological, and functional evaluations. In addition, it is known that persons with an ADL-disability might have an increased risk of being diagnosed with dementia. Aside from their usefulness as a diagnostic tool, the evaluation of ADLs can also serve as an indicator of dementia. Given that only activities which are relevant for an individual are considered may also be useful in non-pharmacological interventions, such as multicomponent rehabilitation programs. Everyday activities should be used mainly for interventions, with an activity-oriented approach relevant to an individual that reflects his/her previous and present interests. By verifying in the BIA which everyday activities are relevant, a list arises of all the activities that can possibly be targeted in activity-oriented interventions. This can be helpful in further exploring the individualized goals for a tailored intervention, for instance in memory clinics.

Finally, the fact that age contributed to the model requires some research. In this study, age also showed a significant, albeit small, effect. This implies a much higher probability of ending up in the AD group as age progresses. This could be expected since age is seen as a risk factor for dementia. Additionally, regarding ADL, it can be expected that, with increasing age, one’s activity capital or repertoire and functionality decreases, regardless of cognitive decline.

### Limitations of the Study

Firstly, a measurement bias might have occurred when assessing ADLs by solely using self-reporting in CHP and using informant reporting in persons with MCI and AD. Though this closely resembles the clinical reality of evaluating everyday activities, this might provide a diffuse picture. However, in previous studies the correlations between self-reporting and proxy reporting were investigated and appeared to be positive [21]. Moreover, both methods of evaluating ADLs could be biased due to mood status, social desirability, diminished awareness, denial, or cognitive deficits [18,39,40]. Additionally, considering informant reporting, informants may vary in their actual contact with the patients and in their capabilities to provide valid information. Secondly, this study relies on a rather small sample and exploratory analysis, meaning that the results are sensitive to small changes and no strong conclusions can be drawn. Thirdly, another potential bias is that the CHP were ‘apparently’ cognitively healthy, meaning that it is still possible that mild cognitive problems were present in some of them. However, we use strict cutoffs of MMSE which can be considered as a valuable instrument for cognitive screening in order to rule out cognitive deficits. Moreover, the conceptualization of MCI itself is tricky since a consensus on its definition and standardized assessment is still lacking. The differentiation between cognitively healthy, MCI, and mild AD is challenging no matter what assessment domain is considered.

## 5. Conclusions

Based on the existing evidence it might be recommended that ADLs are assessed in a stepwise and semi-structured approach. The core aim is to objectify the perception of the patient and his/her relatives with regard to performing ADLs, as recommended by Devi [41]. What can be advised is to explore one’s daily live and meaningful activities, not by using ‘one size fits all’ questionnaires, but by structured interviewing to uncover the patient’s life history and their relevant activities. Only relevant activities should be taken into account to establish eventual functional decline. The next step is to explore the quality of performance and, in case of limitations, the underlying cause(s) of these limitations. This method of ADL evaluation needs standardized manuals to enhance its psychometrical soundness. In addition, though this evaluation might seem time-consuming, it is potentially time-saving when considering the entire span of the care process, starting from diagnosis and moving towards treatment and rehabilitation. During diagnosis, an accurate ADL evaluation offers more valid diagnoses when embedded in a multidisciplinary workup. In non-pharmacological treatment and rehabilitation, information from an in-depth ADL evaluation is valuable for use in activity-oriented interventions and multicomponent rehabilitation programs [42]. In clinical practice a tool to evaluate ADLs in an easy and efficient way, by not disturbing patients and not imposing professionals with a huge workload, is needed. Therefore, it can be argued that the BIA, and especially the i-ADL, could serve as a non-invasive, easy-to-administer, non-time-consuming screening tool. The BIA is the first instrument to objectify a decline in functionality at three complexity levels, resolving the cause of impairment. Once the person with functional limitations caused by cognitive problems is identified, a multidomain assessment should be conducted, including the BIA as a whole, and discussed by a multidisciplinary team to distinguish the mild from the moderate and severe disorders. In our opinion, it is utopic to find one single measure that can perfectly predict cognitive diagnosis. Moreover, an integrated multidisciplinary diagnostic approach improves dementia care [43]. This way of working allows for tailored management as a follow-up, and thus excellent dementia care.

## Figures and Tables

**Table 1:** Demographic characteristics, cognitive functioning measured with MMSE and CAMCOG, daily functioning measured with the BIA.

	CHP (*n* = 47)	MCI (*n* = 39)	AD (*n* = 44)	Post Hoc *p*-Values		
				HC vs. MCI	MCI vs. AD	HC vs. AD
Demographics						
Age Mean (±SD) Range	77.94 (5.26) 68–91	82.08 (5.24) 71–96	82.41 (4.80) 74–92	<0.001	<0.001	<0.001
Gender * Female/male (*n*)	26/21	11/28	12/32	NS		
Education, yrs Mean (±SD) Range	12.40 (2.92) 8–16	11.95 (2.88) 6–16	10.52 (2.79) 6–16	NS	NS	.007
Cognition						
MMSE TOTAL (./30) Mean (±SD) Range	28.28 (1.40) 25 ^1^–30	26.96 (1.70) 23–30	22.20 (22.45) 18–27	<0.001	<0.001	<0.001
CAMCOG TOTAL (./105) Mean (±SD) Range	92.94 (4.46) 80–101	85.68 (3.93) 79–95	72.02 (8.93) 47–87	<0.001	<0.001	<0.001
CAMCOG MEMORY (./27) Mean (±SD) Range	21.66 (1.72) 18–25	18.11 (2.98) 12–27	12.82 (3.50) 4–20	<0.001	<0.001	<0.001
ADL Indices b-ADL %						
b-ADL-DI Mean (±SD) Range	2.30 (6.83) 0–42	9.19 (12.29) 0–38	18.66 (17.46) 0–75	0.044	0.003	<0.001
b-ADL-CDI Mean (±SD) Range	0 (0) 0–0	1.83 (4.95) 0–17	6.53 (11.24) 0–54	NS	0.009	<0.001
b-ADL-PDI Mean (±SD) Range	2.30 (6.83) 0–42	7.91 (11.58) 0–38	14.68 (17.25) 0–75	NS	0.047	<0.001
ADL Indices i-ADL %						
i-ADL-DI Mean (±SD) Range	9.47 (9.87) 0–50	35.76 (24.14) 0–86	51.70 (18.05) 6–94	<0.001	<0.001	<0.001
i-ADL-CDI Mean (±SD) Range	1.19 (4.04) 0–25	19.31 (21.19) 0–72	36.09 (19.32) 0–86	<0.001	<0.001	<0.001
i-ADL-PDI Mean (±SD) Range	5.57 (7.89) 0–28	14.26 (16.56) 0–64	20.46 (18.35) 0–83	0.023	NS	<0.001
ADL Indices a-ADL %						
a-ADL-DI Mean (±SD) Range	21.75 (12.13) 3–54	37.63 (21.89) 3–82	56.84 (17.71) 17–98	<0.001	<0.001	<0.001
a-ADL-CDI Mean (±SD) Range	12.20 (16.34) 0–100	30.53 (21.41) 0–75	49.05 (19.40) 12–93	<0.001	<0.001	<0.001
a-ADL-PDI Mean (±SD) Range	7.77 (8.44) 0–33	9.40 (10.80) 0–41	12.12 (12.60) 0–64	NS	NS	NS

Legend: CHP: Cognitively Healthy Persons; MCI: Mild Cognitive Impairment; AD: Alzheimer’s Dementia; MMSE: Mini Mental State Examination; CAMCOG: Cambridge Cognitive Examination); ADL: Activities of Daily Living; b-: basic; i-: instrumental; a-: advanced; DI: Disability Index; CDI: Cognitive Disability Index; PDI; Physical Disability Index; NS: not significant; SD: standard deviation. * chi-square tests, ^1^ = due to low educational level.

**Table 2:** Observed and predicted diagnosis based on the BIA.

		Predicted Diagnosis		
		CHP (*n*/%)	MCI (*n*/%)	AD (*n*/%)
**Observed** **Diagnosis**	CHP (*n* = 47)	**40 (85.10)**	6 (12.76)	1 (2.13)
	MCI (*n* = 39)	11 (28.20)	**9 (23.10)**	19 (48.72)
	AD (*n* = 44)	1 (2.27)	10 (22.73)	**33 (75.00)**

Legend—CHP: Cognitively Healthy Persons; MCI: Mild Cognitive Impairment; AD: Alzheimer’s Dementia. Correctly predicted diagnosis in bold.

## Data Availability

Data supporting the reported results can be received upon request.

## Supplementary Materials

The following are available online at https://www.mdpi.com/article/10.3390/ijerph182111623/s1, Table S1: the items of basic, instrumental, and advanced Activities of Daily Living of the Brussels Integrated Activities of Daily Living Inventory.
