# Supplementary material for: The Usefulness of Evaluating Performance of Activities in Daily Living in the Diagnosis of Mild Cognitive Disorders
Source: Int J Environ Res Public Health. 2021 Nov 5;18(21):11623. doi: 10.3390/ijerph182111623 (PMC8583568; doi:10.3390/ijerph182111623)
Supplement: Supplementary file 1 [file ijerph-18-11623-s001.zip › ijerph-1423571-supplementary.pdf]

Table S1: The items of basic-, instrumental - and advanced Activities of Daily Living of the Brussels Integrated Activities of Daily Living tool.

Basic- Activities of Daily Living items			
**Item according to Katz's Index**	**ICF-activity**	**ICF-code**	**ICF-definition**
Bathing	Washing oneself	d510	Washing and drying one's whole body, or body parts, using water and appropriate cleaning and drying materials or methods, such as bathing, showering, washing hands and feet, face and hair, and drying with a towel.
Dressing	Dressing	d540	Carrying out the coordinated actions and tasks of putting on and taking off clothes and footwear in sequence and in keeping with climatic and social conditions, such as by putting on, adjusting and removing shirts, skirts, blouses, pants, undergarments, saris, kimono, tights, hats, gloves, coats, shoes, boots, sandals and slippers.
Transferring	Indoor Mobility and changing basic body position	d410	Getting into and out of a body position and moving from one location to another, such as getting up out of a chair to lie down on a bed, and getting into and out of positions of kneeling or squatting.
	Transferring oneself	d420	Moving from one surface to another, such as sliding along a bench or moving from a bed to a chair, without changing body position.
	Walking	d450	Moving along a surface on foot, step by step, so that one foot is always on the ground, such as when strolling, sauntering, walking forwards, backwards, or sideways.
Continence	Regulating urination	d5300	Coordinating and managing urination, such as by indicating need, getting into the proper position, choosing and getting to an appropriate place for urination, manipulating clothing before and after urination, and cleaning oneself after urination.
	Regulating defecation	d3501	Coordinating and managing defecation, such as by indicating need, getting into the proper position, choosing and getting to an appropriate place for defecation, manipulating clothing before and after defecation, and cleaning oneself after defecation
Toileting	Toileting	d530	Planning and carrying out the elimination of human waste (urination and defecation), and cleaning oneself afterwards.

Feeding	Eating	d550	Carrying out the coordinated tasks and actions of eating food that has been served, bringing it to the mouth and consuming it in culturally acceptable ways, cutting or breaking food into pieces, opening bottles and cans, using eating implements, having meals, feasting or dining.
	Drinking	d560	Taking hold of a drink, bringing it to the mouth, and consuming the drink in culturally acceptable ways, mixing, stirring and pouring liquids for drinking, opening bottles and cans, drinking through a straw or drinking running water such as from a tap or a spring.

Instrumental Activities of Daily Living			
**Item according to Lawton Scale**	**ICF-activity**	**ICF-code**	**ICF-definition**
Telephone use	Using communication devices and techniques	d360	Using devices, techniques and other means for the purposes of communicating, such as calling a friend on the telephone.
Using transportation	Using transportation	d470	Using transportation to move around as a passenger, such as being driven in a car or on a bus, rickshaw, jitney, animal-powered vehicle, or private or public taxi, bus, train, tram, subway, boat or aircraft.
Shopping	Shopping	d6200	Obtaining, in exchange for money, goods and services required for daily living (including instructing and supervising an intermediary to do the shopping), such as selecting food, drink, cleaning materials, household items or clothing in a shop or market; comparing quality and price of the items required, negotiating and paying for selected goods or services, and transporting goods.
Preparing food	Preparing meals	d630	Planning, organising, cooking and serving simple and complex meals for oneself and others, such as by making a menu, selecting edible food and drink, getting together ingredients for preparing meals, cooking with heat and preparing cold foods and drinks, and serving the food.
Housekeeping	Doing housework	d640	Managing a household by cleaning the house, washing clothes, using household appliances, storing food and disposing of garbage, such as by sweeping, mopping, washing counters, walls and other surfaces; collecting and disposing of household garbage; tidying rooms, closets and drawers; collecting, washing, drying, folding and ironing clothes; cleaning footwear; using brooms, brushes and vacuum cleaners; using washing machines, driers and irons.

Doing laundry	Washing and drying clothes	d6400	Washing clothes and garments and hanging them out to dry in the air.
Doing handyman work	Caring for household objects	d650	Maintaining and repairing household and other personal objects, including house and contents, clothes, vehicles and assistive devices, and caring for plants and animals, such as painting or wallpapering rooms, fixing furniture, repairing plumbing, ensuring the proper working order of vehicles, watering plants, grooming and feeding pets and domestic animals.
Responsibility for own medications	Maintaining one's health	d5702	Caring for oneself by being aware of the need and doing what is required to look after one's health, both to respond to risks to health and to prevent ill-health, such as by seeking professional assistance; following medical and other health advice; and avoiding risks to health such as physical injury, communicable diseases, drug-taking and sexually transmitted diseases.
Handling finance	Basic economic transitions	d860	Engaging in any form of simple economic transaction, such as using money to purchase food or bartering, exchanging goods or services; or saving money.

Advanced Activities of Daily Living			
**ICF - activities Cluster**	**ICF - code**	**ICF - definition**	**Items**
Sophisticated kitchen activities	d6301	Advanced cooking, complex meals with a large number of ingredients, using complex methods of preparation or making dinner with several courses; baking bread, cakes	Freezing or pickling vegetables
			Baking bread, cakes
			Cooking complex meals
			Try out new dishes
			Making jam
Household appliances and daily technology	d6403	The use of electronically equipment inside and outside the house, including reading and understanding manuals	Using Magnetron
			Using Dish washer
			Using Oven
			Using Coffee machine
			Using Kitchen aid
			Using Washing machine
			Using Drying machine
			Using Radio / CD
			Using TV
			Using Video / DVD

			Using Camera
			Using Lawn mower
			Using Electric saw
			Using High pressure cleaner
			Using manuals explaining daily technology
High level gardening	d6505	To cultivate vegetables and special or rare plants	/
Cognitive stimulating activities or intellectual activities	d166 & d9200	Playing games, reading books, etc…; to read professional literature, books and magazines in other languages, use of computer programs, use of an agenda	Playing puzzles and brainteasers
			Using PC programs
			Using internet
			Using agenda
			Reading books
			Reading professional or educational literature, other languages
			Writing books, poems, articles

Craftwork and arts	d6500 & d9203	Knitting, sewing, repairing clothes, reattaching buttons and fasteners; practicing arts like painting, sculpturing and others, playing music instruments	Crafting<br>Playing music instrument<br>Practicing arts
Complex economic activities or transactions	d865	To be involved in some form of complex economic transactions like trading in commodities, the use of bank cards, 'money out the wall' system, PC-banking	Electronically banking, to pay electronically, to use money out of the wall system<br>Complex administration and banking
To communicate by using devices or techniques	d360	The use of cell phones, corresponding through email	Using a cell phone<br>Writing a mail or a letter
Sports	d9201	To be engage in informal or organized sports: group activities and sporting on your own, e.g. fishing, ride a bicycle	Doing sports<br>Riding bicycle
Transportation by motorized vehicles	d475	To drive a car, motorcycle	/
Self development/self realization/self educational activities	d9202 & d 810	To develop oneself by formal or informal learning: attending a course, going to lectures, consuming arts (visiting exhibitions, musical performances)	/

To go on a holiday	d920	Going on holiday, in an own cottage or participating in group trips	/
Caring for or assisting others	d660 & d6506	To care for household members (mostly the partner), often by helping to handle medication, helping with bathing, dressing or assisting in transfers; or caring for (grand)children and to provide help in household tasks, to take care of pets, by feeding and cleaning them and exercising them	Helping (in the business of) the children<br>Taking care of partner<br>Taking care of (great) grand children<br>Taking care of pets
Caring for household objects	d560	Activities like painting, wallpapering rooms, fixing furniture, plumbing in the own place or in that of others	/
Semi-professional work	d855	To work as a volunteer, engaged in non-remunerative employment and performing 'semi-professional work': social jobs, administration, accountancy, often as a continuation of one's profession	/
Engagement in organized social live or leisure activities	d910 & d9250	Active participation in organized communities or societies by taking part in meetings, being member of the board, organizing activities for others or by participating in activities organized by others, like short trips and coffee moments; to be engaged in forms of activity only for amusement or relaxation, like to go out for dinner with partner, children, friends and to	Organising events<br>Making and keeping appointments<br>Taking part in meetings, conversations

		visit family. All activities clustered in this category encompass a social factor by doing things just for the fun of being together, socializing	
